# Forecasting the Suitability of Online Mental Health Information for Effective Self-Care Developing Machine Learning Classifiers Using Natural Language Features

**DOI:** 10.3390/ijerph181910048

**Published:** 2021-09-24

**Authors:** Meng Ji, Wenxiu Xie, Riliu Huang, Xiaobo Qian

**Affiliations:** 1 School of Languages and Cultures, University of Sydney, Sydney 2006, Australia; rhua5035@uni.sydney.edu.au; 2 Department of Computer Science, City University of Hong Kong, Hong Kong 518057, China; Vasiliky@outlook.com; 3 School of Computer Science, South China Normal University, Guangzhou 510631, China; xiaoboqian1221@outlook.com

**Keywords:** mental health, machine learning, information actionability, mental self-care support

## Abstract

Background: Online mental health information represents important resources for people living with mental health issues. Suitability of mental health information for effective self-care remains understudied, despite the increasing needs for more actionable mental health resources, especially among young people. Objective: We aimed to develop Bayesian machine learning classifiers as data-based decision aids for the assessment of the actionability of credible mental health information for people with mental health issues and diseases. Methods: We collected and classified creditable online health information on mental health issues into generic mental health (GEN) information and patient-specific (PAS) mental health information. GEN and PAS were both patient-oriented health resources developed by health authorities of mental health and public health promotion. GENs were non-classified online health information without indication of targeted readerships; PASs were developed purposefully for specific populations (young, elderly people, pregnant women, and men) as indicated by their website labels. To ensure the generalisability of our model, we chose to develop a sparse Bayesian machine learning classifier using Relevance Vector Machine (RVM). Results: Using optimisation and normalisation techniques, we developed a best-performing classifier through joint optimisation of natural language features and min-max normalisation of feature frequencies. The AUC (0.957), sensitivity (0.900), and specificity (0.953) of the best model were statistically higher (*p* < 0.05) than other models using parallel optimisation of structural and semantic features with or without feature normalisation. We subsequently evaluated the diagnostic utility of our model in the clinic by comparing its positive (LR+) and negative likelihood ratios (LR−) and 95% confidence intervals (95% C.I.) as we adjusted the probability thresholds with the range of 0.1 and 0.9. We found that the best pair of LR+ (18.031, 95% C.I.: 10.992, 29.577) and LR− (0.100, 95% C.I.: 0.068, 0.148) was found when the probability threshold was set to 0.45 associated with a sensitivity of 0.905 (95%: 0.867, 0.942) and specificity of 0.950 (95% C.I.: 0.925, 0.975). These statistical properties of our model suggested its applicability in the clinic. Conclusion: Our study found that PAS had significant advantage over GEN mental health information regarding information actionability, engagement, and suitability for specific populations with distinct mental health issues. GEN is more suitable for general mental health information acquisition, whereas PAS can effectively engage patients and provide more effective and needed self-care support. The Bayesian machine learning classifier developed provided automatic tools to support decision making in the clinic to identify more actionable resources, effective to support self-care among different populations.

## 1. Introduction

The determinants of mental health disorders are complex [1]. Potential contributors to mental health may be classified into three large categories: first, individual attributes, which include low self-esteem, cognitive, emotional immaturity, difficulties in communicating, medical illness, and substance use [2] second, social circumstances, which include loneliness, bereavement, neglect, family conflict, exposure to violence and abuse, low income and poverty, difficulties or failure at school, work stress and unemployment [3,4,5]; third, environmental factors, which encompasses poor access to basic services, injustice, discrimination, social, gender inequalities, and exposure to war or disaster [6,7,8,9]. These adverse factors may interact and compound one another to significantly increase the vulnerability of an individual to mental disorders. Given the diversity of determinants of mental health issues, protective measures proposed for mental health issues at individual, social and environmental levels also vary. For individuals with personal traits, which make an individual more vulnerable to mental disorders, support includes building self-esteem, self-confidence, improvement of communicative skills, and enhancing abilities to solve practical problems and manage self-stress [10,11,12,13]. For individuals suffering from lack of employment or study-related stresses, support measures include economic security, improved satisfaction, and success at work. For individuals whose mental disorders are caused by social, environmental factors, policy interventions are required to increase equality of access to basic services, social justice, tolerance, integration, and gender equality.

Despite the diversity in mental health determinants and needs for more diverse support mechanisms to vulnerable populations, most current public-oriented mental health information is general patient information (GEN). GEN refers to health resources that have been adapted from professional medical resources for the public. It is significantly less complex than specialised information for health and medical professionals. General patient information is often developed by health agencies and health promotion organisations. Our study focuses on personalised online mental healthcare information developed for population segments of known vulnerability to mental health disorders, especially young people. Research shows that the development of personalised mental healthcare information can increase motivation, highlight risks, change attitudes, and counterbalance the lack of personal contact in computerised health interventions [14]. Personalised mental health information requires the identification of practical, varying needs of certain populations such as young adults, recognising their strengths, addressing the social determinants of mental health, educational and employment opportunities causing mental health disorders among young people, and helping them to achieve their potential for better mental health and wellbeing. 

Machine learning is playing an increasingly important role in prompting and facilitating personalised mental healthcare [15,16,17,18]. It is being used to develop machine learning models to predict and detect mental diseases such as Alzheimer’s disease and other forms of dementia, as well as the progression of mental health conditions among populations at high risks such as vulnerable young people. The combination of natural language processing techniques and machine learning is gaining momentum in developing digital language-based, written, or spoken interventions to support personalised mental healthcare through web-based or smartphone apps [19,20,21,22,23]. In our study, we utilised machine learning to detect mental health information, which was developed using a more personalised, people-centred communicative style compared with the more general, abstract health information on depression and anxiety caused by personal, social, and environmental determinants. The machine learning classifier we developed produces Bayesian probabilistic output, the likelihood of a certain online mental health message as being suitable for young people needing personalised mental healthcare information. It can be used to enhance the online information search experiences among young people with mental health issues and help them achieve optimal online mental healthcare outcomes.

## 2. Methods

### 2.1. Data Collection

We collected and classified these personalised mental healthcare resources as patient-tailored mental health information (PAS). They were purposefully developed for patients and their caregivers based on the age profiles and vulnerability of mental health patients with a focus on young people. PAS information provides an effective strategy to increase the understandability and, more importantly, the actionability of health information, as PAS mostly provides the target readers with useful, real-life-based self-healthcare information through narratives of lived experiences of people with similar mental health conditions. We searched for PAS information on the websites of credible websites developed by national or state health agencies and not-for-profit organisations in the UK (9 websites), Australia (5 websites), and Canada (9 websites). Given that different health agencies and organisations use distinct information labelling strategies, the database we developed contained mental healthcare information for diverse young age groups: young people, young people aged 11–18, children and teenager, children and young people under the age of 35, 11–16-year-olds, young under 35, youth and young adults 14–29, young people 18–25, young people 12–24, youth, children and youth, and young men. 

### 2.2. Feature Annotation

Feature annotation used Readability Studio (Oleander Software) [24,25] and an automatic semantic annotation system (USAS) developed by University of Lancaster in the UK [26,27,28]. Readability Studio added 27 natural language features regarding morphological, lexical, and syntactic complexity of the texts: medical jargons, number of unique words, repeated words, article mismatches, wording errors, redundant phrases, overused words, wordy items, cliché, number of proper nouns, number of numerals, average number of characters, average number of syllables, number of monosyllabic words, number of unique monosyllabic words, number of complex (3+ syllable) words, number of unique 3+ syllable words, number of long (6+ characters) words, number of unique long words, average number of sentences per paragraph, number of difficult sentences (more than 22 words), average sentence length, passive voice, sentences that begin with conjunctions, number of interrogative sentences (questions), and number of exclamatory sentences. USAS added 22 large semantic categories: general/abstract terms (A), medicine/health (B), arts and culture (C), emotion (E), food (F), government (G), dwelling (H), employment (I), sports (K), living things (L), locations (M), measurements (N), general substances (O), education (P), speech acts (Q), social actions (S), time (T), environment (W), psychology (X), science/tech (Y), names and grammar (Z), and out of dictionary (Z99). The total number of features was 49.

### 2.3. Classifier Training and Testing 

We divided the entire corpus of mental healthcare information into training (70%) and validation datasets (30%). The training and validation datasets contained 559 and 241 articles on patient-specific mental healthcare (focusing on young people), and 701 and 299 articles on generic mental healthcare, respectively. This mirrors the proportion between these two sets of mental healthcare samples collected, which was 4:5. Training data were then divided into 5 folds of equal sizes for cross-validation to reduce bias in the model training and testing stages. In each iteration, 4 folds of data were used to train the model with the remaining fold to test the model. This procedure was repeated 5 times so that each fold was used as test data once. Lastly, we used the left-out 30% data to validate the model and assess its performance on new data after training and testing through the 5-fold cross-validation procedure.

### 2.4. Bayesian Machine Learning Classifiers (BMLC)

In health document classification, support vector machine (SVM) has been widely used as a highly efficient supervised learning technique. Its key features include good generalisation performance to avoid model overfitting, fast computing speed, and model simplicity as the output of SVM is a linearly weighted sum of nonlinear, fixed basis functions. The disadvantages or limitations of SVM are also known: its model complexity normally increases as the training size grows; SVM outputs are hard, with dichotomous class membership rather than probabilistic outcomes; there are requirements for cross-validation of training datasets to estimate the best trade-off between minimal classification errors and the separating margin between two classes being predicted. Relevance vector machine (RVM) is the Bayesian version of SVM [29,30,31,32]. It does not suffer from any of the above-mentioned shortcomings of SVM. RVM is a highly sparse classifier with good generalisability, even with small training datasets in hundreds. A distinct feature of RVM, which made it to the top of our learning models list, is its probabilistic output of class membership as a function of varying class priors and asymmetric misclassification costs, that is, false negative and false positive costs. Posterior probabilities allow us to capture uncertainty or adjustability of outcomes in the prediction.

### 2.5. Bayesian Probabilistic Assessment of Personalised Mental Healthcare Quality

In our study of classification, the prediction of mental health selfcare information in terms of its actionability for vulnerable people and RVM output in the form of posterior probability of class membership can help us understand 3 important, interrelated, yet understudied aspects of patient mental health information design: First, the impact of the prior probability of a certain mental healthcare text at the predefined actionability level (PAS vs. GNE) on the posterior probability of the binary prediction of mental information actionability. In our study, the actionability of a certain mental healthcare information is assessed through the website information labelling strategy: either designed for certain target population (young people) or the general public. Patient-specific mental healthcare information was defined as information of high actionability, as their contents were well designed for certain target readers, helping them to effectively implement mental healthcare recommendations. By contrast, generic patient mental healthcare information was defined as information of low actionability, as their contents are more general, abstract, focusing on standard medical and healthcare support measures without specific recommendations for a certain target vulnerable population group. Second, the impact of the distribution of a range of structural and semantic features in mental healthcare information on the actionability of the resources. Through building Bayesian machine learning classifiers based on separate or combined structural and semantic features, we attempted to capture the relations between complex linguistic profiles of mental healthcare information in the training dataset and the prediction outcomes. Developing linguistically interpretable Bayesian classifiers can provide useful guidelines to the development of mental healthcare information suitable for the target populations.

Third, the impact of probability thresholds on the posterior probabilities of the prediction of outcomes. Although 0.5 is an intuitive probability cut-off, in practical settings, this is often decided by the desired sensitivity-specificity pairing. Sensitivity and specificity are inversely proportional, that is, as sensitivity increases, specificity decreases and vice versa. In our study, the primary research aim was to separate mental healthcare information of high actionability from those of low actionability. A higher sensitivity means that the classifier can detect a larger proportion of online mental healthcare information of low actionability from those of high actionability, and a higher specificity means that the classifier is able to detect a larger percentage of mental healthcare information of high actionability from those of low actionability. Higher sensitivity is often associated with higher false positive rate, which means more information that is suitable for the targeted audience would be misclassified as information of low-actionability, potentially causing more human costs to review and revise the documents unnecessarily. Higher negativity is associated with higher false negative rate, which means that more information that is not suitable for the target audience would be misclassified as suitable and actionable mental health information. A model of high false negativity can severely undermine AI-assisted mental healthcare information assessment, especially when the intended readers are vulnerable young people. When weighing the benefits and risks of models of varying sensitivity and specificity pairs, it would be more desirable to choose those of higher specificities in our study, when the sensitivity levels are also acceptable for the available resources and staffing capacity.

### 2.6. Classifier Optimisation

**Figure 1:**
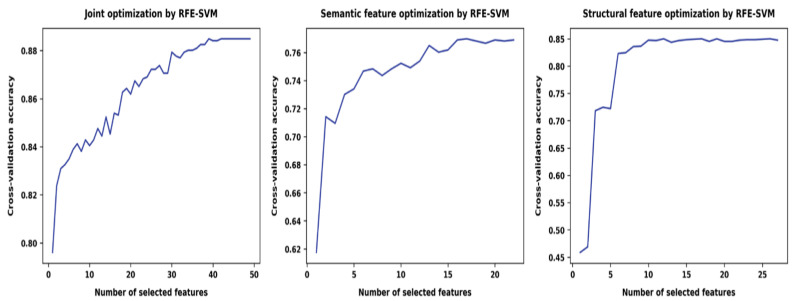
Automatic feature elimination RFE on different feature sets.

Similar to its non-probabilistic version SVM, the relevance vector machine (RVM) is a sparse classifier that performs better with a limited number of features. Reducing the number of features can help better identify the separating surface between binary classes, thus improving the accuracy of the algorithm. To explore optimised feature sets from the multiple features we added based on existing research from health linguistics and readability assessment in medical education, we first applied automatic feature selection techniques using cross-validation accuracy (1 minus minimal classification errors MCE) as the selection criterion. The higher the cross-validation accuracy, the more accurate the model based on a certain feature set. The feature deduction used was recursive feature elimination with SVM as the base estimator. Figure 1 shows the results of the automatic feature selection from different sets of features including semantic features (middle), structural features (right), and the combination of both feature sets (left).

The cross-validation classification accuracy of automatic feature optimisation on semantic features was 0.77. The optimised semantic feature set reduced original semantic features from 22 to 17, representing a 22.7% reduction rate: medicine/health (B), emotion (E), government (G), dwelling (H), employment (I), sports (K), living things (L), locations (M), measurements (N), general substances (O), time (T), environment (W), psychology (X), science/tech (Y), names and grammar (Z), and out of dictionary (Z99). The cross-validation classification accuracy of automatic feature optimisation on structural features was 0.85. The optimised structural feature set reduced the original structural features from 27 to 12, a 45% reduction rate: morphological features (reduced from 8 to 3 features): average number of characters, average number of syllables, number of unique 3+ syllable words; lexical features (reduced from 10 to 3 features): medical jargons, wordy items, and cliché; and syntactic features (reduced from 9 to 6): number of difficult sentences (more than 22 words), number of interrogative sentences (questions), number of exclamatory sentences, passive voice, sentences that begin with conjunctions, and sentences that begin with lowercased words.

Finally, the cross-validation classification accuracy of automatic feature optimisation on both semantic and structural features was 0.885. Joint optimisation reduced the original features from 49 to 39, representing 79.59% of the original structural and semantic feature sets—morphological features (8 unchanged): number of unique words, average number of characters, number of monosyllabic words, number of unique monosyllabic words, number of complex (3+ syllable) words, number of unique 3+ syllable words, number of long (6+ characters) words, and number of unique long words; lexical features (reduced from 10 to 5): number of numerals, number of proper nouns, misspellings, and overused words, wordy items; syntactic features (reduced from 9 to 7): average number of sentences per paragraph, number of difficult sentences, average sentence length, number of interrogative sentences, number of exclamatory sentences, sentences beginning with conjunctions, and sentences that begin with lowercased words; and semantic features (reduced from 22 to 19): general/abstract terms (A), medicine/health (B), arts and culture (C), emotion (E), government (G), dwelling (H), sports (K), living things (L), locations (M), measurements (N), general substances (O), speech acts (Q), social actions (S), time (T), environment (W), psychology (X), science/tech (Y), names and grammar (Z), and out of dictionary (Z99).

### 2.7. Feature Normalisation and Scaling

Feature normalisation has proved an effective technique to improve the accuracy of machine learning classifiers such as SVM in the detection of risks and diseases. Min-max is a popular normalisation technique. It applies a linear transformation of the original data and recasts the original feature values to the range of 0 and 1. To improve the accuracy of models running on both different original and optimised feature sets, we used min-max normalisation to transfer the original feature values and then assessed the impact of normalisation on the model performance (Table 1).

## 3. Results

Table 1 compares the performance of 6 sets of RVM classifiers. Through comparing their performance on the training/testing and validation datasets, we aimed to identify the best-performing classifier. The first set (i) contains 3 classifiers with non-optimised and non-normalised features. In the classifier training and testing stages, the mean AUC of RVM with non-optimised, non-normalised semantic features (SOF22) was 0.858 (SD = 0.022) and was the lowest compared with that of RVM with non-optimised, non-normalised structural features (TOF27) (mean AUC = 0.926, SD = 0.018) and that of RVM with both sets’ features (SOF22, TOF27), which did not undergo any optimising and scaling treatment (mean AUC = 0.960, SD = 0.013). The AUC of RVMs of these 3 classifiers improved in the validation from 0.858 to 0.875 (SOF22), 0.926 to 0.927 (TOF27), and 0.960 to 0.965 (SOF22 + TOF27). The second set (ii) contains the normalised version of these 3 classifiers. Feature normalisation improved their performance consistently: the mean AUC of the 3 RVM improved from 0.858 to 0.861 (SOF22), 0.926 to 0.953 (TOF27), and 0.9560 to 0.962 (SOF22 + TOF27). The performance of classifiers in the validation stage also improved consistently. This suggests that normalisation improved the accuracy of classifiers with different feature sets. The third set of classifiers (iii) was based on separately optimised semantic features (SOF17) and structural features (TOF12), as well as their combination (SOF17 + TOF12). The mean AUC of RVM with these optimised, non-normalised feature sets in the training/testing stage was slightly lower than that of their counterparts with non-optimised, non-normalised feature sets (i). This suggested that separate optimisation and the subsequent combinations did not improve the performance of classifiers. The fourth feature set (iv) contains the normalised version of classifiers in (iii). Again, normalisation improved the performance of classifiers, but their performance was still lower than that of their counterparts (ii), which were normalised, non-optimised features. Next, we conducted joint optimisation of the entire feature set containing semantic (SOF22) and structural (TOF27) features, and the result was shown in (v). Joint optimisation proved a more efficient feature selection method: first, the mean AUC of the jointly optimised classifier largely remained the same as the non-optimised, non-normalised classifier (0.960); second, the number of features reduced from 49 to 39, including 20 structural and 19 semantic features, increasing the generalisability and interpretability of the model. To further improve the performance of the jointly optimised classifier, we normalised the classifier and increased the mean AUC to 0.966 (SD = 0.011).

**Figure 2:**
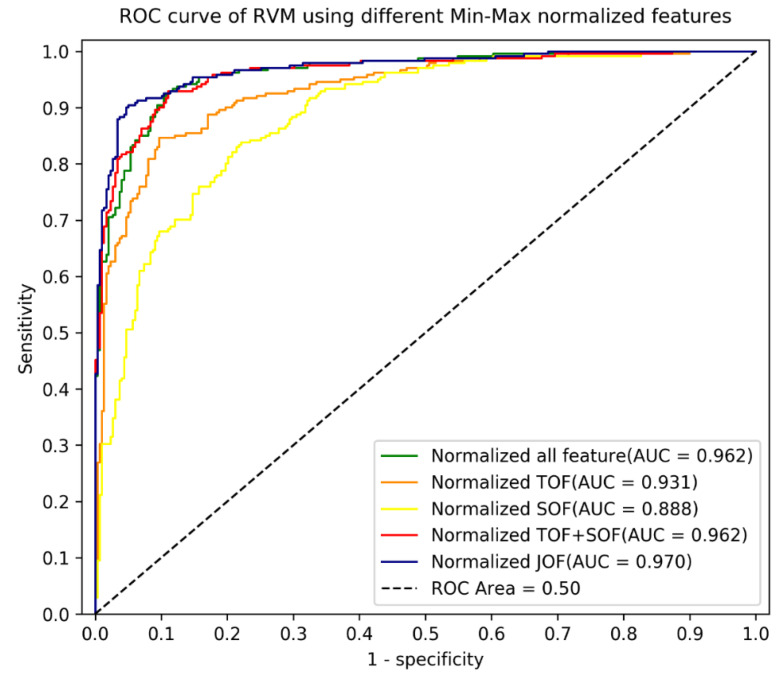
Receiver operating characteristic (ROC) of RVM of normalised, optimised features on validation data.

Table 2 and Figure 2 show that the jointly optimised and normalised classifier achieved the highest mean AUC (0.966). This was statistically higher than that of RVM with normalised semantic features (SOF22) (*t* test, AUC_ _mean difference_ = 0.1048, 95% CI: 0.037, 0.173, *p* = 0.0024), RVM with normalised structural features (TOF27) (AUC_ _mean difference_ = 0.040, 95% CI: 0.009, 0.070, *p* = 0.0046), RVM with optimised and normalised semantic features (SOF17) (AUC_ _mean difference_ = 0.101, 95% CI: 0.053, 0.150, *p* = 0.0008), RVM with optimised and normalised structural features (TOF12) (AUC_ _mean difference_ = 0.025, 95% CI: 0.02, 0.033 *p* = 0.0002), and RVM with separately optimised and then combined, normalised features (SOF17 + TOF12) (AUC_ _mean difference_ = 0.015, 95% CI: 0.007, 0.023, *p* = 0.0014).

Table 3 shows Mann–Whitney U test of differences in sensitivity and specificity between the jointly optimised and normalised classifier (JOF39) and other normalised and optimised feature sets. Sensitivity of RVM_JOF (39) was statistically higher than that of RVM based on non-optimised, non-normalised structural features TOF (27) (*p* = 0.022); RVM based on non-optimised, normalised structural features TOF (27) (*p* = 0.022); RVM of non-optimised, non-normalised sematic features SOF (22) (*p* = 0.022); RVM based on non-optimised, normalised semantic features TOF (27) (*p* = 0.012); RVM with non-normalised, optimised structural features TOF (12) (*p* = 0.012); RMV with normalised, optimised structural features TOF (12) (*p* = 0.012); RVM with non-normalised, optimised semantic features SOF (17) (*p* = 0.021); RMV with normalised, optimised semantic features SOF (12) (*p* = 0.012); RVM with non-normalised, separately optimised, and then combined features (SFO17 + TOF12) (*p* = 0.022); and RVM with normalised, separately optimised, and then combined features (SFO17 + TOF12) (*p* = 0.046). Sensitivity of RVM with jointly optimised and normalised features (JOF39), however, did not improve when compared to the original full feature set (SOF22 + TOF27) (*p* = 0.074) and its normalised version (*p* = 0.346). This, however, achieved the desired effect of feature reduction without compromising the performance of the classifier.

Similarly, specificity of RVM_JOF (39) was statistically higher than that of RVM based on non-optimised, non-normalised structural features TOF (27) (*p* = 0.022); RVM based on non-optimised, normalised structural features TOF (27) (*p* = 0.022); RVM of non-optimised, non-normalised sematic features SOF (22) (*p* = 0.047); RVM based on non-optimised, normalised semantic features TOF (27) (*p* = 0.012); RVM with non-normalised, optimised structural features TOF (12) (*p* = 0.012); RMV with normalised, optimised structural features TOF (12) (*p* = 0.028); RVM with non-normalised, optimised semantic features SOF (17) (*p* = 0.012); RMV with normalised, optimised semantic features SOF (12) (*p* = 0.012); and RVM with non-normalised, separately optimised, and then combined features (SFO17 + TOF12) (*p* = 0.022). Specificity of RVM with jointly optimised and normalised features (JOF39) did not improve when compared to RVM with normalised, separately optimised, and then combined features (SFO17 + TOF12) (*p* = 0.095), as well as RVM with the original full features (SOF22 + TOF27) (*p* = 0.249) and its normalised version (*p* = 0.144).

## 4. Discussions

### 4.1. Probabilistic Outputs

**Figure 3:**
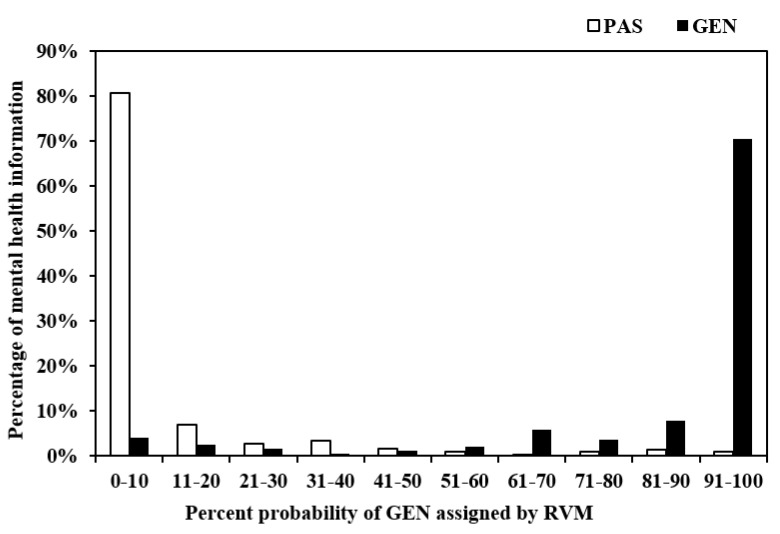
Percentage of generic (GEN) and patient-specific (PAS) mental health texts assigned to each 10% probability bin.

A major strength of Bayesian machine learning classifiers such as RVMJ is their probabilistic outputs instead of hard binary prediction as SVM. These are posterior probabilities of class membership, which are adaptive to varying prior probabilities and conditional probabilities. Our study used natural language features to classify and predict the likelihood of a certain piece of online mental healthcare information of being actionable and effective for young people with mental health issues. In this context, conditional probability may be interpreted as the probability of the text being classified or predicted as actionable effect, or not, given the probability of the occurrence of certain natural language features, structural or semantic. Probabilistic output enables a more intuitive interpretation of the classifier output than the outcome converted to a non-linear scale through postprocessing. In the research and practice of patient-oriented mental health information evaluation, probabilistic output, as shown in Figure 3, can help clinicians, researchers, and health educators to make more informative decisions of the suitability, actionability, and usability of a certain mental healthcare for the target readers.

Figure 3 shows the assignment of the two sets of mental healthcare information, generic and patient-specific, to each of the 10% probability bins by the best-performing classifier based on the jointly optimised and normalised feature set (JOF39). It shows that 95.32% of patient-specific mental healthcare texts were assigned to probability bins ranging from 0–10% to 41–50%. This was the specificity of the model; 90.04% of generic mental healthcare information was assigned to probability bins in the range of 51–60% to 91–100%. This was the sensitivity of the model. Less than 5% of generic mental healthcare information was misclassified as suitable and actionable for the target readers (false-negative cases), and around 10% of high-quality young-people-specific mental healthcare resources were misclassed as non-suitable for this reader group (false positive cases). Depending on practical needs, sensitivity and specificity of the model could be further adjusted to achieve the desired sensitivity and specificity pair by varying the probability thresholds, as discussed below.

### 4.2. Diagnostic Utility of Bayesian Classifiers to Predict Mental Healthcare Information Suitability for Young People

We examined the performance of the best performing RVM classifier on the validation data using sensitivity, specificity, area under the curve of the receiver operator curve, and accuracy. In the assessment of clinical tools, two other useful indicators are positive likelihood ratio and negative likelihood ratio. Positive likelihood ratio (LR+) is calculated by divide sensitivity (true positive rate) with 1 minus specificity (false positive rate); and negative likelihood ratio (LR−) is calculated by divide 1 minus sensitivity (false negative rate) with specificity (true negative rate) ] [33,34,35,36]. The larger the positive likelihood ratio, the higher the posterior probability of a test result of being positive for the case is truly positive; and the smaller the negative likelihood ratio, the lower the posterior probability of a test result being negative when the case is truly positive, or, in other words, the higher the posterior probability of a test being negative when the case is truly negative. The advantage of positive and negative likelihood ratios is that they are not affected by disease prevalence or the prior probability of a health condition being tested [36,37,38]. In the context of our study, this can be translated as: for machine learning classifiers (equivalents to clinical testing tools) with positive likelihood ratios, the posterior probabilities of positive prediction are higher for mental health resources that are generic and non-suitable for the target young readers. Moreover, for machine learning classifiers with lower negative likelihood ratios, the posterior probabilities of negative prediction are higher for patient-specific, actionable mental health information for the intended information user groups. A good classifier should combine high sensitivity, high specificity, high positive likelihood ratio (10 to be acceptable in the clinic), and low negative likelihood ratio (under 0.1 to be acceptable in the clinic).

Table 4 shows that sensitivity, specificity, positive likelihood ratio (LR+), and negative likelihood ratio (LR−) of the best performing classifier with increasing thresholds ranging between 0.1 and 0.9. It is intuitive to set the threshold at 0.5, that is, texts with posterior probabilities of 0.5 or above, which were classified as generic mental healthcare information, were non-suitable for young people; and texts with posterior probabilities of smaller than 0.5 as patient-specific mental healthcare information were suitable for young people with mental health issues. However, in practice, interpretation of this table and identification of the best threshold is largely dependent on the desired function of the evaluation tool. If we raised the threshold, classifier sensitivity and LR− decreased as specificity and LR+ increased. This means that increasing probability threshold would lead to less false positive and more false negative predictions. If we lowered the threshold, sensitivity increased as specificity, and LR+ and LR− decreased. This means that decreasing probability threshold would lead to more false-positive and less false-negative predictions. Decision needs to be based on the cost-effectiveness of the classifier and its clinical utility. The best threshold was suggested for 0.4, which was associated with a high sensitivity (0.913), high specificity (0.936), and clinically acceptable LR+ (14.366) and LR− (0.093). Increasing the threshold would render the evaluation tool less safe to use in the clinic, as a higher false negative rate would lead to more generic, less actionable mental healthcare information to be misclassified as suitable information for the readers. Decreasing the threshold would reduce the cost-effectiveness of the classifier, as a higher false positive rate would result in more unnecessary human expert review and revision when the materials were suitable for young readers. This would cause the dilemma of developing low-cost machine learning classifiers to reduce human expert workload in less well-resourced working environments.

## 5. Conclusions

Patient-centred mental healthcare information has significant advantage over generic mental health information regarding information actionability, engagement, and suitability for specific populations with distinct mental health issues. Generic mental health information is more suitable for general mental health information acquisition, whereas patient-centred mental healthcare information can effectively engage patients and provide more effective and needed self-care support. The Bayesian machine learning classifier we developed provided automatic tools to support decision making in the clinic to identify more actionable resources, effective to support self-care among different populations. Developing probabilistic machine learning classifiers can provide useful, highly precise, adaptive, and intuitive decision aids towards personalised, patient-centred mental healthcare information evaluation. Our study made a useful attempt in this promising direction.

### Limitations

The training data we collected were primarily intended for young people with mental health issues. More research is needed to extend the generalisability of the classifier for other user groups, as well as its applicability in resources written in other languages.

## Figures and Tables

**Table 1:** Performance of RVM with different feature sets on training/testing and validation data.

Technique	Training/Testing (5-Fold Cross-Validation)	Validation			
	AUC Mean (SD)	AUC	Accuracy	Sensitivity	Specificity
**(i) Non-Optimised, Non-Normalised Feature Sets**					
RVM_ SOF (22)	0.858 (0.022)	0.875	0.774	0.747	0.796
RVM_ TOF (27)	0.926 (0.018)	0.927	0.848	0.805	0.883
RVM_ SOF (22) + TOF (27)	0.960 (0.009)	0.965	0.9	0.8589	0.933
**(ii) Non-Optimised, Normalised Feature Sets**					
RVM_ normalised SOF (22)	0.861 (0.038)	0.886	0.793	0.730	0.843
RVM_ normalised TOF (27)	0.953 (0.019)	0.939	0.869	0.805	0.920
RVM_ normalised SOF (22) + TOF (27)	0.963 (0.013)	0.962	0.889	0.851	0.920
**(iii) Separate Optimisation without Normalisation**					
RVM_ SOF (17)	0.858 (0.026)	0.870	0.785	0.755	0.809
RVM_ TOF (12)	0.906 (0.026)	0.887	0.802	0.739	0.853
RVM_ SOF (17) + TOF (12)	0.943 (0.013)	0.940	0.867	0.830	0.896
**(iv) Separate Optimisation with Normalisation**					
RVM_ normalised SOF (17)	0.864 (0.029)	0.888	0.8	0.734	0.853
RVM_ normalised TOF (12)	0.941 (0.010)	0.931	0.874	0.838	0.903
RVM_ normalised SOF (17) + TOF (12)	0.951 (0.012)	0.962	0.898	0.863	0.926
**(v) Joint Optimisation without Normalisation**					
RVM_ JOF (39)	0.960 (0.008)	0.965	0.893	0.851	0.926
**(vi) Joint Optimisation with Normalisation**					
RVM_ normalised JOF (39)	0.966 (0.011)	0.970	0.9296	0.9004	0.953

**Table 2:** Pair-wise corrected resampled t-test of area under the curve (AUC) of RVMs on the training/testing data.

Pairs	RVM Classifier Pair(s)	AUC Mean Difference	Asymptotic 95% Confidence Interval		
			Lower	Upper	*p*-Value
1	RVM_ normalised JOF (39) vs. RVM_ normalised SOF (22)	0.105	0.037	0.172	0.0024 **
2	RVM_ normalised JOF (39) vs. RVM_ normalised SOF (17)	0.101	0.053	0.150	0.0008 **
3	RVM_ normalised JOF (39) vs. RVM_ normalised TOF (27)	0.040	0.009	0.070	0.0046 **
4	RVM_ normalised JOF (39) vs. RVM_ normalised TOF (12)	0.025	0.02	0.033	0.0002 **
5	RVM_ normalised JOF (39) vs. RVM_ normalised SOF (12) + TOF (17)	0.015	0.007	0.023	0.0014 **

** statistical significane at 0.05 level.

**Table 3:** Mann–Whitney U test of differences in sensitivity and specificity of classifiers on validation data.

Pair	Mann–Whitney U Test	Sensitivity	Asymptotic 95% C.I.			Specificity	Asymptotic 95% C.I.		
		Mean Difference	Lower	Upper	*p*-Value	Mean Difference	Lower	Upper	*p*-Value
1	RVM_ normalised JOF (39) vs. RVM_ TOF (27)	0.073	0.009	0.137	0.022 **	0.083	0.034	0.132	0.022 **
2	RVM_ normalised JOF (39) vs. RVM_ normalised TOF (27)	0.079	0.006	0.152	0.022 **	0.037	0.019	0.056	0.047 **
3	RVM_ normalised JOF (39) vs. RVM_ SOF (22)	0.113	−0.022	0.248	0.022 **	0.181	0.071	0.292	0.012 **
4	RVM_ normalised JOF (39) vs. RVM_ normalised SOF (22)	0.135	0.000	0.269	0.012 **	0.122	0.056	0.189	0.012 **
5	RVM_ normalised JOF (39) vs. RVM_ TOF (12)	0.148	0.083	0.213	0.012 **	0.109	0.057	0.162	0.012 **
6	RVM_ normalised JOF (39) vs. RVM_ normalised TOF (12)	0.066	0.029	0.104	0.012 **	0.062	0.031	0.092	0.028 **
7	RVM_ normalised JOF (39) vs. RVM_SOF (17)	0.114	−0.036	0.263	0.021 **	0.189	0.055	0.322	0.012 **
8	RVM_ normalised JOF (39) vs. RVM_ normalised SOF (17)	0.128	0.016	0.240	0.012 **	0.126	0.033	0.220	0.012 **
9	RVM_ normalised JOF (39) vs. RVM_ SOF (17) + TOF (12)	0.068	−0.001	0.137	0.022 **	0.063	0.025	0.100	0.022 **
10	RVM_ normalised JOF (39) vs. RVM_ normalised SOF (17) + TOF (12)	0.034	0.010	0.058	0.046 **	0.023	0.004	0.043	0.095
11	RVM_ normalised JOF (39) vs. RVM_ SOF (22) + TOF (27)	0.034	0.006	0.062	0.074	0.021	0.007	0.035	0.249
12	RVM_ normalised JOF (39) vs. RVM_ normalised TOF (27) + SOF (22)	0.025	−0.024	0.074	0.346	0.026	0.005	0.048	0.144

** statistical significane at 0.05 level.

**Table 4:** Thresholds, positive/negative likelihood ratio, 95% CI of the best performing RVM classifier on the test data.

Probability Thresholds	Sensitivity (95% CI)	Specificity (95% CI)	Positive Likelihood Ratio (LR+) (95% CI)	Negative Likelihood Ratio (LR−) (95% CI)
0.1	0.959 (0.933, 0.984)	0.806 (0.761, 0.851)	4.941 (3.916, 6.235)	0.051 (0.028, 0.095)
0.2	0.934 (0.902, 0.965)	0.876 (0.839, 0.914)	7.545 (5.570, 10.220)	0.076 (0.047, 0.122)
0.3	0.917 (0.882, 0.952)	0.903 (0.869, 0.937)	9.455 (6.676, 13.389)	0.092 (0.060, 0.140)
0.4	0.913 (0.877, 0.948)	0.936 (0.909, 0.964)	14.366 (9.281, 22.236)	0.093 (0.062, 0.140)
0.45	0.905 (0.867, 0.942)	0.950 (0.925, 0.975)	18.031 (10.992, 29.577)	0.100 (0.068, 0.148)
0.5	0.900 (0.863, 0.938)	0.953 (0.929, 0.977)	19.230 (11.512, 32.125)	0.104 (0.071, 0.153)
0.55	0.884 (0.843, 0.924)	0.960 (0.938, 0.982)	22.022 (12.627, 38.407)	0.121 (0.085, 0.172)
0.6	0.880 (0.839, 0.921)	0.963 (0.942, 0.985)	23.911 (13.369, 42.785)	0.125 (0.089, 0.176)
0.7	0.822 (0.773, 0.870)	0.967 (0.947, 0.987)	24.565 (13.318, 45.309)	0.185 (0.141, 0.242)
0.8	0.784 (0.732, 0.836)	0.977 (0.959, 0.994)	33.498 (16.061, 69.864)	0.221 (0.174, 0.281)
0.9	0.705 (0.648, 0.763)	0.990 (0.979, 1.001)	70.304 (22.737, 217.388)	0.298 (0.245, 0.362)

Machine learning classifier: RVM classifier (with min-max normalised JOF39).

## Data Availability

Not applicable.
